# Status asthmaticus

**DOI:** 10.1038/s41390-025-04177-9

**Published:** 2025-05-30

**Authors:** Tina S. Chai

**Affiliations:** Vanderbilt University School of Medicine, Nashville, TN USA

## Abstract

The poem examines the tension between medical detachment, particularly in critical care settings, and familial grief using hyper-specific medical language contrasting against the warmth of the mother and grandmother. The poem also reflects on how quickly life shifts from routine to crisis. The piece adds a humanized perspective of care to literature. In ICUs, patients are often reduced to the systems that fail them. This problem-by-organ-system model is efficient for clinicians but can erode patients’ personhood. The piece hopes to spark discussion about medical systems that may simplify patients to organ systems, particularly in critical care settings.

**Status asthmaticus**

Mom said you wheezed all your life, sweet and low,

and that morning was like any other:

Lucky Charms at 7:00 —

Except when you coughed it up,

milky brown wads glazed in pink and green,

a chewed-up rainbow and half a pot of gold

Then slid to the ground, silent and slow,

as if accepting surrender,

your head resting on marble tile

While Grandma keened

*Sam, Sam, Sam*

like it was the only word she knew

As your lungs replied with gasps,

chest with rubbery heaves,

your heart thud to a pause

And started again

but your mind had quieted

no longer playing *To Pimp a Butterfly* on repeat

Now the ventilator hums in C,

your mouth agape in a hollow note

singing your final song.

